# Adiposity and hand osteoarthritis: the Netherlands Epidemiology of Obesity study

**DOI:** 10.1186/ar4447

**Published:** 2014-01-22

**Authors:** A Willemien Visser, Andreea Ioan-Facsinay, Renée de Mutsert, Ralph L Widya, Marieke Loef, Albert de Roos, Saskia le Cessie, Martin den Heijer, Frits R Rosendaal, Margreet Kloppenburg

**Affiliations:** 1 Department of Rheumatology, C1-R, Leiden University Medical Center, P.O. Box 9600, 2300 RC Leiden, the Netherlands; 2 Department of Clinical Epidemiology, C7-P, Leiden University Medical Center, Box 9600, 2300 RC Leiden, the Netherlands; 3 Department of Radiology, C2-S, Leiden University Medical Center, P.O. Box 9600, 2300 RC Leiden, the Netherlands; 4 Department of Endocrinology, Vrije Universiteit Medical Center, P.O. Box 7057, 1007 MB Amsterdam, the Netherlands

## Abstract

**Introduction:**

Obesity, usually characterized by the body mass index (BMI), is a risk factor for hand osteoarthritis (OA). We investigated whether adipose tissue and abdominal fat distribution are associated with hand OA.

**Methods:**

The Netherlands Epidemiology of Obesity (NEO) study is a population-based cohort aged 45 to 65 years, including 5315 participants (53% women, median BMI 29.9 kg/m^2^). Fat percentage and fat mass (FM) (kg) were estimated using bioelectrical impedance analysis. The waist-to-hip ratio (WHR) was calculated. In 1721 participants, visceral adipose tissue (VAT) and subcutaneous adipose tissue (SAT) (cm^2^) were assessed using abdominal MR imaging. Hand OA was defined according to the ACR criteria.

Odds ratios (OR) with 95% confidence intervals (CI) were calculated for the association of fat percentage, FM, WHR, VAT and SAT with hand OA using logistic regression analyses per standard deviation, stratified by sex and adjusted for age.

**Results:**

Hand OA was present in 8% of men and 20% of women. Fat percentage was associated with hand OA in men (OR 1.34 (95% CI 1.11 to 1.61)) and women (OR 1.26 (1.05 to 1.51)), as was FM. WHR was associated with hand OA in men (OR 1.45 (1.13 to 1.85)), and to a lesser extent in women (OR 1.17 (1.00 to 1.36)). Subgroup analysis revealed that VAT was associated with hand OA in men (OR1.33 (1.01 to 1.75)). This association increased after additional adjustment for FM (OR 1.51 (1.13 to 2.03)).

**Conclusions:**

Fat percentage, FM and WHR were associated with hand OA. VAT was associated with hand OA in men, suggesting involvement of visceral fat in hand OA.

## Introduction

Osteoarthritis (OA) is the most common musculoskeletal disorder. Although the pathogenesis of OA remains largely unknown, several risk factors are known to contribute to disease development. One of the most prominent risk factors is overweight or obesity, usually defined by body mass index (BMI) of 25 to 30 kg/m^2^ or ≥30 kg/m^2^, respectively [1]. Obesity acts as a risk factor for both weight-bearing joints and nonweight-bearing joints, suggesting that obesity-associated systemic factors could play an important role in OA [2,3]. The relative contribution of systemic factors and excessive biomechanical stress in the association between obesity and OA remains to be elucidated and could be different for different subtypes of OA, such as hand OA versus knee OA.

Although the systemic effects of obesity are most probably dependent on the amount and distribution of adipose tissue, most studies on OA performed until now used BMI as marker for obesity. However, since BMI is defined based only on height and weight, it provides little information about body composition and the amount and distribution of adipose tissue. More insight into the relation between adiposity and OA can be obtained when alternative measures of body composition are investigated, such as the fat percentage, fat mass (FM) and waist-to-hip ratio (WHR). Previous research assessing these body composition measures mostly studied knee OA and showed inconclusive results regarding FM [4-9], whereas WHR was not associated with OA [4,7,10]. Only a few studies focused on OA in nonweight-bearing joints such as the hands, showing no association with fat percentage and waist circumference and conflicting results regarding the WHR [8,11-13].

Adipose tissue is a source of several cytokines that could influence whole-body metabolism. Secretion of these bioactive mediators depends on the type of adipose tissue; they are secreted more actively by visceral fat than by subcutaneous fat [14]. In addition, visceral fat has been shown to be associated more strongly with obesity-related co-morbidities, such as diabetes mellitus and the metabolic syndrome, and with markers of inflammation as compared with subcutaneous fat [15,16]. Cytokines have the potential to affect joint tissues [17-19], and therefore visceral fat could also be involved in the pathogenesis of OA. No research has so far been performed regarding different body fat depots in relation to OA.

The aim of the present study was to gain more insight into the mechanisms underlying the association of adiposity and OA. To this end, we investigated the association of adipose tissue and its abdominal distribution with the presence of OA in nonweight-bearing joints, the hands.

## Methods

### Study design and study population

The Netherlands Epidemiology of Obesity (NEO) study is a population-based prospective cohort study in lean, overweight and obese individuals aged between 45 and 65 years. The present study is a cross-sectional analysis of the baseline measurements of the 5,313 participants included in the NEO study between September 2008 and January 2012. Detailed information about the study design and data collection has been described previously [20]. Men and women with self-reported BMI ≥27 kg/m^2^ living in the greater area of Leiden (in the west of the Netherlands) were eligible to participate in the NEO study. In addition, in one municipality (Leiderdorp) all inhabitants aged 45 to 65 years were invited, irrespective of their BMI. All participants completed questionnaires on demographic and clinical data and visited the NEO study center for several baseline measurements. The study was approved by the medical ethics committee of the Leiden University Medical Center and all participants gave written informed consent.

### Body composition measures

Measured body weight (kg) and height (cm) were used to calculate the BMI (kg/m^2^). Waist and hip circumference (cm) were used to calculate the WHR. The percentage of body fat and amount of FM (kg) were measured by bioelectrical impedance analysis (BIA) using the Tanita foot-to-foot BIA system TBF-300A Body Composition Analyzer (Tanita Corporation of America, Inc, Arlington Heights, IL, USA) [21]. To test the reliability, repeated measurements were performed after approximately 3 months in a random sample of 72 participants; the calculated intraclass correlation coefficient was 0.98.

### Abdominal adipose tissue

A random sample (about 30%) of the study participants without contraindications (metallic devices, claustrophobia and a body circumference ≥170 cm) underwent magnetic resonance imaging (MRI) of the abdomen. Abdominal visceral adipose tissue (VAT) and subcutaneous adipose tissue (SAT) (cm^2^) were measured by a turbo spin echo imaging protocol, performed on a 1.5 T system (Philips, Medical Systems, Best, the Netherlands): echo time, 11 milliseconds; repetition time, 168 milliseconds; flip angle, 90°; slice thickness, 10 mm. The total acquisition time, including the initial survey sequence, was 3 minutes. At the level of the fifth lumbar vertebra, three transverse images with a slice thickness of 10 mm were obtained during a breath-hold. The MASS software package (Medis, Leiden, the Netherlands) was used to quantify VAT and SAT, allowing a semi-automated detection of the VAT and SAT area. The mean values of VAT and SAT (cm^2^) were calculated.

### Osteoarthritis definition

Self-reported pain was measured using standardized questionnaires. Physical examination of the hand joints was performed by trained research nurses, using a standardized scoring form. Bony and soft swellings of the distal interphalangeal (DIP), proximal interphalangeal (PIP), metacarpophalangeal (MCP), carpometacarpal (CMC) and wrist joints were scored, as well as deformities of the DIP, proximal interphalangeal (PIP), first metacarpophalangeal (MCP), carpometacarpal (CMC) and wrist joints. OA was defined according to the criteria of the American College of Rheumatology as pain or stiffness on most days of the prior month in addition to three of the following criteria: bony swelling of ≥2 of the 10 selected joints (bilateral DIP II and III, PIP II and III, and first CMC joints), bony swelling of ≥2 DIP joints, <3 swollen MCP joints, and deformity of ≥1 of the 10 selected joints [22].

### Statistical analysis

Data were analyzed using SPSS version 20 (SPSS, Chicago, IL, USA) and STATA version 12 (StataCorp LP, College Station, TX, USA).

In the NEO study there is an oversampling of persons with BMI ≥27 kg/m^2^. To correctly represent associations in the general population [23], adjustments were made for the oversampling of individuals with BMI ≥27 kg/m^2^. This was done by weighting individuals towards the BMI distribution of participants from the Leiderdorp municipality [24], whose BMI distribution was similar to the BMI distribution in the general Dutch population [25]. Consequently, results apply to a population-based study without oversampling of BMI ≥27 kg/m^2^.

Pearson correlation coefficients between all body composition measures were calculated. A correlation below 0.4 was considered weak, between 0.4 and 0.7 moderate, and above 0.7 strong. Logistic regression analyses were used to calculate cross-sectional associations of all body composition measures with hand OA, and were expressed as odds ratios (ORs) with 95% confidence intervals (CIs). All continuous variables were standardized by dividing individual values by the standard deviation to be able to compare the ORs, because in this way all ORs describe the effect on the odds of OA of an increase of one standard deviation of the corresponding variable. All analyses have been stratified by sex and adjusted for age. To minimize variation in FM due to differences in body height, additional adjustment for height was performed in the analysis on FM. Furthermore, additional adjustment for FM has been performed in the analyses on visceral and subcutaneous fat in relation to hand OA.

## Results

### Population characteristics

After exclusion of subjects with missing data for the BIA (*n* = 25) or physical examination (*n* = 4), data from 5,284 subjects were analyzed. Table 1 presents the baseline characteristics. Median age was 56 years, and 53% were women. Women had a lower median body weight and WHR but a higher fat percentage and FM than men. Hand OA was present in 8% of men and 20% of women.

**Table 1:** Baseline characteristics of the total Netherlands Epidemiology of Obesity study population and stratified by sex

	**Total population**	**Men**	**Women**
	**(*n*** **= 5,284)**	**(*n*** **= 2,490)**	**(*n*** **= 2,794)**
Age (years)	56 (51 to 61)	57 (51 to 61)	56 (51 to 61)
BMI (kg/m^2^)	29.9 (27.8 to 32.8)	29.6 (27.9 to 32.0))	30.3 (27.8 to 33.5)
Weight (kg)	90.6 (80.6 to 100.8)	96.6 (89.2 to 106.0)	84.0 (75.8 to 94.2)
Fat percentage (%)	37.5 (29.0 to 43.7)	29.0 (25.9 to 32.7)	43.3 (39.9 to 46.4)
Fat mass (kg)	32.4 (26.1 to 40.0)	28.1 (23.6 to 34.0)	36.4 (30.4 to 43.1)
WHR	0.93 (0.87 to 0.99)	0.98 (0.94 to 1.03)	0.88 (0.84 to 0.92)
VAT (cm^2^)^a^	122.1 (85.4 to 166.3)	142.1 (108.1 to 185.5)	97.2 (67.8 to 138.2)
SAT (cm^2^)^a^	308.3 (242.3 to 388.4)	262.4 (210.3 to 324.2)	359.4 (298.7 to 434.0)
Hand osteoarthritis	746 (14.1)	188 (7.6)	558 (20.0)

Numbers represent medians (interquartile ranges) or number (percentage).

BMI, body mass index; SAT, subcutaneous adipose tissue; VAT, visceral adipose tissue; WHR, waist-to-hip ratio.

^a^*n* = 923 men, *n* = 798 women.

Abdominal fat was measured in a random subset of 1,721 participants (46% women). Except for a clinically nonrelevant difference in WHR in men (0.980 vs. 0.982), this subgroup did not differ from the total group (data not shown).

The median amount of VAT was lower than the median amount of SAT, and this difference was most apparent in women (Table 1).

### Correlations between body composition measures

First, we calculated Pearson’s correlation coefficients between all measures of body composition (Table 2). Besides body weight, BMI was strongly correlated with fat percentage and FM in both men and women. BMI, body weight, fat percentage and FM were strongly correlated with SAT in both sexes. The correlations of these body composition measures with VAT were somewhat lower, and were slightly stronger in women as compared with men. Moreover, WHR correlated more strongly with VAT than with SAT in both sexes. The WHR showed a stronger correlation with all measurements of fat (FM, SAT and VAT) in men as compared with women.

**Table 2:** Correlations between body composition measures in 2,490 men (right upper corner) and 2,794 women (left lower corner) of the Netherlands Epidemiology of Obesity study

	**BMI (kg/m**^**2**^**)**	**Weight (kg)**	**Fat percentage (%)**	**Fat mass (kg)**	**WHR**	**VAT**^**a**^** (cm**^**2**^**)**	**SAT**^**a **^**(cm**^**2**^**)**
**BMI (kg/m**^**2**^**)**		0.864	0.859	0.920	0.608	0.667	0.802
**Weight (kg)**	0.919		0.727	0.893	0.480	0.564	0.787
**Fat percentage (%)**	0.871	0.890		0.949	0.656	0.696	0.753
**Fat mass (kg)**	0.927	0.981	0.951		0.612	0.679	0.825
**WHR**	0.490	0.462	0.526	0.493		0.683	0.512
**VAT**^**a **^**(cm**^**2**^**)**	0.738	0.691	0.720	0.727	0.595		0.479
**SAT**^**a **^**(cm**^**2**^**)**	0.873	0.849	0.846	0.878	0.448	0.623	

All correlations were statistically significant. Numbers represent Pearson correlation coefficients.

BMI, body mass index; SAT, subcutaneous adipose tissue; VAT, visceral adipose tissue; WHR, waist-to-hip ratio.

^*a*^*n* = 923 men, *n* = 798 women.

The differences between men and women underscored the need for stratified analyses in the sexes.

### Associations of body composition measures with hand osteoarthritis

We next investigated the associations of all body composition measures with hand OA (Table 3).

**Table 3:** Associations of body composition measures and hand osteoarthritis

	**Standard deviation**		**Adjusted odds ratio (95% CI)**	
	**Men**	**Women**	**Men (*n*** **= 2,490)**	**Women (*n*** **= 2,794)**
Fat percentage (%)	6.22	6.88	1.34 (1.11 to 1.61)	1.26 (1.05 to 1.51)
Fat mass (kg)	9.39	10.76	1.24 (1.05 to 1.47)	1.22 (1.07 to 1.39)
WHR	0.07	0.07	1.45 (1.13 to 1.85)	1.17 (1.00 to 1.36)
BMI (kg/m^2^)	4.01	5.19	1.29 (1.08 to 1.55)	1.25 (1.11 to 1.41)
Weight (kg)	14.49	14.73	1.15 (0.92 to 1.45)	1.20 (1.06 to 1.36)

All odds ratios express the increase in odds of osteoarthritis per standard deviation and are adjusted for age.

BMI, body mass index; CI, confidence interval; WHR, waist-to-hip ratio.

The fat percentage was associated with hand OA in both men (OR = 1.34 (95% CI = 1.11 to 1.61)) and women (OR = 1.26 (95% CI = 1.05 to 1.51)), meaning that one standard deviation increase in fat percentage (men 6.22%, women 6.88%) is associated with a 34% higher risk of having hand OA in men and a 26% higher risk in women. FM was also associated with hand OA in both sexes (men: OR = 1.24 (95% CI = 1.05 to 1.47); women: OR = 1.22 (95% CI = 1.07 to 1.39)). Additional adjustment for height in the analysis on FM resulted in comparable ORs (men: OR = 1.29 (95% CI = 1.10 to 1.51); women: OR = 1.25 (95% CI = 1.10 to 1.42)).

When focusing on the distribution of adipose tissue, we observed the WHR to be associated with hand OA in men (OR = 1.45 (95% CI = 1.13 to 1.85)) and to a lesser extent in women (OR = 1.17 (95% CI = 1.00 to 1.36)).

### Abdominal adipose tissue

Since both the amount of adipose tissue and its distribution are of importance, we investigated the associations of VAT and SAT with hand OA in a subgroup who underwent MRI of the abdomen (Table 4). No association was observed between SAT and hand OA. VAT, on the other hand, showed a statistically significant association with hand OA in men (OR = 1.33 (95% CI = 1.01 to 1.75)) but was not associated with hand OA in women.

**Table 4:** Associations of abdominal adipose tissue and hand osteoarthritis

	**Standard deviation**		**Adjusted odds ratio (95% CI)**	
	**Men**	**Women**	**Men (*n*** **= 923)**	**Women (*n*** **= 798)**
VAT (cm^2^)	61.3	50.0	1.33 (1.01 to 1.75)	1.10 (0.85 to 1.44)
SAT (cm^2^)	91.7	117.6	1.05 (0.74 to 1.50)	1.22 (0.92 to 1.63)

All odds ratios express the increase in odds of osteoarthritis per standard deviation and are adjusted for age.

CI, confidence interval; SAT, subcutaneous adipose tissue; VAT, visceral adipose tissue.

Since VAT and SAT are associated with the total amount of body fat, we also assessed their association with hand OA independent of total body fat by additional adjustment for FM. As a result, the association of VAT with hand OA in men increased (OR = 1.51 (95% CI = 1.13 to 2.03)). In women, again no association between VAT and hand OA was observed (OR = 0.91 (95% CI = 0.69 to 1.20)).

## Discussion

In this study we aimed to gain insight into the association between adiposity and hand OA. Since both the fat percentage and FM were associated with hand OA in men and women, the amount of adipose tissue seems to be important. The association between WHR and hand OA indicates that the fat distribution is also of importance. When assessing the abdominal distribution of adipose tissue, VAT was shown to be associated with hand OA in men, suggesting involvement of visceral fat in hand OA.

To our knowledge, this study is the first to show an association between the amount of fat and its abdominal distribution with hand OA. Other studies showed associations between OA of the hands and obesity-related co-morbidities: Jonsson and colleagues demonstrated that hand OA and atherosclerosis were associated in older women; both carotid plaques and coronary calcifications showed a linear association with hand OA severity [26]. Hoeven and colleagues confirmed this observation in a population aged 55 years and older; they showed an association of atherosclerosis and OA of the DIP and MCP joints in women, independent of cardiovascular risk factors [27]. Finally, Haara and colleagues showed that symmetrical DIP OA predicted mortality in women and that OA in any finger joint predicted cardiovascular mortality in men, suggesting an underlying common metabolic factor [28].

A possible common underlying explanation could be an effect of adipose tissue, especially the visceral component. Visceral fat has been shown previously to be associated with coronary calcifications and carotid atherosclerosis [29,30]. The amount of visceral fat has also been associated with other obesity-related co-morbidities such as diabetes mellitus and metabolic risk factors such as elevated blood pressure, impaired fasting glucose and elevated triglycerides [15,31,32]. Our study shows that visceral fat is also associated with hand OA, implying that adipose tissue and its products can be involved in hand OA.

Visceral fat has been suggested to secrete bioactive cytokines, acting as a unique pathogenic fat depot [14]. The involvement of visceral fat in the pathogenesis of hand OA might thus be explained by its secretion of cytokines, which have been suggested to act locally in joint tissues [17]. Leptin, known especially for its proinflammatory effect, has been shown to affect human cartilage [17-19]. Adiponectin appears to counteract the effect of leptin by anti-inflammatory actions [17]. *In vitro* studies suggest that adiponectin affects chondrocyte function and modulates cartilage destruction, which might indicate a protective role for adiponectin in OA [33]. This suggestion has been confirmed in an observational follow-up study in patients with hand OA, showing that a higher level of adiponectin is associated with a lower risk for hand OA progression [34]. Knowledge on other adipose-derived cytokines in relation to OA is scarce.

Differences between both sexes regarding body compositions are well known and were also observed in this study. Women had a lower WHR, more subcutaneous fat and less visceral fat than men. The WHR was more strongly correlated to all measurements of fat in men than in women. This is in accordance with previous studies describing sex differences in body composition measures [35,36]. Because of these differences between men and women regarding most body composition measures, all analyses were stratified by sex.

The greater amount of overall fat and lower susceptibility to accumulate visceral fat in women as compared with men might explain the lower ORs of WHR and VAT for hand OA in women. A similar gender difference regarding VAT has been described previously in a study on cardiometabolic risk; VAT was observed to be of greater relevance in men, whereas total FM was of most importance in women [37]. In addition, VAT was observed to be associated with insulin resistance and inflammatory markers primarily in men [38,39]. Another explanation for the lower ORs of WHR and VAT in women might be the importance of unmeasured or unknown risk factors such as hormonal status or genetic effects, overshadowing a possible relatively minor effect of visceral fat.

There are some potential limitations of this study. Hand OA could only be diagnosed based on clinical criteria since no imaging data of the hands were available. However, the ACR clinical criteria are well validated and have a high sensitivity and specificity in diagnosing hand OA [22].

Furthermore, the fat percentage and FM were measured using a foot-to-foot BIA system, and not with a hand-to-foot BIA. Although it has been suggested that foot-to-foot BIA might overestimate the amount of FM [40], a study comparing body fat percentages provided by foot-to-foot BIA with those obtained by hand-to-foot BIA observed a strong correlation between the two methods (*r* = 0.84) [21]. In a study comparing resistance measurements obtained from foot-to-foot BIA with those from underwater weighing and dual-energy X-ray absorptiometry, a strong correlation (*r* = 0.89) with both methods was also reported [41].

We investigated all body composition measures in relation to hand OA per standard deviation to be able to compare the different ORs observed in this study. However, whereas the fat percentage and FM involve whole body fat, the amounts of VAT and SAT apply to a small region of the abdominal fat depot. The ORs for fat percentage and FM therefore cannot be compared directly with the ORs for VAT and SAT.

The amount of VAT and SAT were measured in a random 30% of the total study population. Although individuals with a body circumference of 170 cm or higher were not eligible for MRI, body composition measures of the MRI subgroup were not significantly different as compared with the total study population. However, since individuals with extremely high body circumference could not be assessed, the described association between VAT and hand OA might be underestimated.

## Conclusion

This study showed that both the amount of adipose tissue and its distribution are of importance in hand OA. Assessment of the abdominal distribution of adipose tissue showed an association between VAT and hand OA in men, suggesting involvement of visceral fat in hand OA. More research is necessary to gain more insight into the role of adipose tissue in OA, aiming at abdominal fat distribution and secretion of cytokines in relation to OA. Longitudinal studies could help to better understand how visceral fat plays a role in OA development. Furthermore, research towards treatment aiming at the inflammatory effect of adipose tissue may lead to potential new treatment targets in OA.

## Abbreviations

BIA: bioelectrical impedance analysis; BMI: body mass index; CI: confidence interval; CMC: carpometacarpal; DIP: distal interphalangeal; FM: fat mass; MCP: metacarpophalangeal; MRI: magnetic resonance imaging; NEO: Netherlands Epidemiology of Obesity; OA: osteoarthritis; OR: odds ratio; PIP: proximal interphalangeal; SAT: subcutaneous adipose tissue; VAT: visceral adipose tissue; WHR: waist-to-hip ratio.

## Competing interests

The authors declare that they have no competing interests.

## Authors’ contributions

AWV performed the statistical analysis, interpreted the data and drafted the manuscript. RLW and ML contributed to the acquisition of the data. AI-F, RdM, AdR, SlC, MdH and FRR participated in the study design. AI-F, RdM, RLW, ML, AdR, SlC, MdH and FRR revised the manuscript for important intellectual content. MK participated in the study design, was involved in analyzing and interpreting the data, and was involved in drafting and revising the manuscript. All authors read and approved the final version of the manuscript.
